# Real-world experience with CDK4/6 inhibitors in hormone receptor-positive metastatic and recurrent breast cancer: findings from an Asian population

**DOI:** 10.1007/s10238-024-01458-1

**Published:** 2024-08-12

**Authors:** Bo-Fang Chen, Yi-Fang Tsai, Ta-Chung Chao, Pei-Ju Lien, Yen-Shu Lin, Chin-Jung Feng, Yen-Jen Chen, Han-Fang Cheng, Chun-Yu Liu, Jiun-I Lai, Ling-Ming Tseng, Chi-Cheng Huang

**Affiliations:** 1 Division of Breast Surgery, Department of Surgery, Taipei Veterans General Hospital, 201, Section 2, Shih-Pai Road, Taipei, 112 Taiwan; 2 Comprehensive Breast Health Center, Department of Surgery, Taipei Veterans General Hospital, Taipei, Taiwan; 3 School of Medicine, National Yang-Ming Chiao-Tung University, Taipei, Taiwan; 4 Faculty of Medicine, School of Medicine, National Yang-Ming Chiao-Tung University, Taipei, Taiwan; 5 Division of Cancer Prevention, Department of Oncology, Taipei Veterans General Hospital, Taipei, Taiwan; 6 Division of Plastic and Reconstructive Surgery, Department of Surgery, Taipei Veterans General Hospital, Taipei, Taiwan; 7 Division of Medical Oncology, Department of Oncology, Taipei Veterans General Hospital, Taipei, Taiwan; 8 Center of Immuno-Oncology, Department of Oncology, Taipei Veterans General Hospital, Taipei, Taiwan; 9 Institute of Clinical Medicine, School of Medicine, National Yang-Ming Chiao-Tung University, Taipei, Taiwan; 10 Institute of Epidemiology and Preventive Medicine, School of Public Health, College of Public Health, National Taiwan University, Taipei, Taiwan

**Keywords:** CDK4/6 inhibitors, Breast cancer, Real-world data, Treatment outcomes, Taiwanese

## Abstract

**Purpose:**

Cyclin-dependent kinase 4/6 inhibitors (CDK4/6i) combined with endocrine therapy have demonstrated significant clinical benefits in progression-free and overall survival. This study investigates the outcomes associated with two kinds of CDK4/6i in patients with hormone receptor (HR)-positive metastatic and relapsed breast cancer to inform real-world evidence of treatment strategies.

**Methods:**

This retrospective study included 340 Taiwanese patients with HR-positive advanced breast cancer from the Taipei Veterans General Hospital, between 2018 and 2023. We analyzed patient characteristics, treatment strategies and outcomes associated with two CDK4/6i. The efficacy of patients who experienced economic burden and interrupted CDK4/6i treatment after 2 years of National Health Insurance (NHI) reimbursement was also investigated.

**Results:**

Patients receiving ribociclib and palbociclib showed no significant differences in age, histology, body mass index(BMI), or pathologic status. The distribution of disease status and endocrine therapy partners was comparable between the two groups. Dose reduction was similar, while patients with palbociclib tended to discontinue CDK4/6i usage, and those with ribociclib tended to switch to the other CDK4/6i or endocrine partners. There was no significant difference in progression-free survival (PFS) between the two CDK4/6i in the first-line setting. Adverse prognostic factors were increasing HER2 IHC score, higher Ki-67 levels, visceral and liver metastasis, prior chemotherapy, and endocrine therapy resistance, while higher BMI, bone-only metastasis, and letrozole treatment were associated with a lower risk of progression. The limited follow-up time in our study was insufficient to assess the outcomes of patients treated with interrupted CDK4/6i for up to two years under the NHI reimbursement policy.

**Conclusion:**

Treatment outcomes between the two types of CDK4/6i did not differ significantly, indicating the safety and efficacy of CDK4/6i for the Asian population. Ribociclib and palbociclib showed similar efficacy in PFS in the real-world setting.

## Background

Cyclin-dependent kinase 4/6 inhibitors (CDK4/6i) combined with endocrine therapy have shown significant clinical benefits in terms of progression-free survival (PFS) and overall survival (OS) for hormone receptor (HR)-positive advanced breast cancer. Randomized controlled trials (RCTs), including PALOMA, MONALEESA, and MONARCH series for palbociclib, ribociclib, and abemaciclib, have demonstrated that CDK4/6i combined with endocrine therapy is superior to endocrine therapy alone [1–3]. Since receiving approval from the Food and Drug Administration (FDA) in 2015, CDK4/6i treatment has become widespread worldwide and the standard of care for HR-positive advanced breast cancers.

While RCTs are considered as the gold standard for evaluating the efficacy and safety of a novel medication due to their strict study design, enrolled criteria, standardized evaluation, and specific patient populations, they have limitations in terms of patient diversity and may not fully represent actual clinical practice efficacy [4]. Real-world data (RWD) play an important role in providing additional evidence for clinical practice, especially in heterogeneous populations [5]. Recent RWD studies for CDK4/6i, such as P-REALITY X for palbociclib and RIBANNA and CompLEEment-1 study for ribociclib, have contributed valuable insights [6–8]. However, most RCTs have enrolled small numbers of Asian patients, and only a few RWD studies have focused on the Asian population [9]. Additionally, due to the timeline of FDA approval, many RWD studies have predominantly included palbociclib usage over ribociclib or abemaciclib when considering all types of CDK4/6i. In Taiwan, under the National Health Insurance (NHI), only palbociclib and ribociclib are reimbursed for HR-positive metastatic breast cancer (mBC) patients currently, resulting in the majority receiving palbociclib and ribociclib rather than abemaciclib.

Despite the known similarities in mechanism and structure, there is a lack of information on the efficacy, adverse effects, and treatment outcomes across distinct CDK4/6i. This study aims to investigate two different CDK4/6i (palbociclib and ribociclib) in patients with HR-positive mBC and relapsed breast cancer (rBC) and provide real-world evidence of safety and treatment strategies in the Asian population. Furthermore, according to the NHI policy in Taiwan, patients with luminal mBC can receive only 2 years of CDK4/6i. Consequently, some patients experienced economic burden and interrupted CDK4/6i treatment after 2 years of reimbursement. We also provided preliminary results regarding the efficacy of treatment after two years in this subpopulation.

## Methods

### Study population and treatments

This observational and retrospective study enrolled patients with HR-positive mBC or rBC patients who underwent treatment of either palbociclib or ribociclib between January 2019 and December 2023 at the Taipei Veterans General Hospital, a tertiary referral medical center in North Taiwan. Patients treated with abemaciclib as the first-line CDK4/6i, as well as those with other types of cancers or early-stage breast cancer, were excluded. Clinical treatment practices adhered to the guidelines established by the Comprehensive Breast Health Center at the Taipei Veterans General Hospital, which were based on recommendations from the National Comprehensive Cancer Network (NCCN), European Society for Medical Oncology (ESMO), and St. Gallen International Consensus guidelines [10–12]. All clinicopathological information, disease status, breast cancer-related prescriptions, and adverse effects were extracted from the Big Data Center and electronic medical records of the Taipei Veterans General Hospital. All potentially identifying patient information will be encrypted, anonymized, deleting names, addresses, and other identifiable items, to provide information security. The study protocols were approved by the Institutional Review Board (IRB-TPEVGH No.: 2024-04-014AC) with waivers of informed consent.

### Data collections and outcomes

HR-positive (luminal) breast cancer was defined as those with estrogen receptor (ER)-positive, with ≥ 1% of tumor cells exhibiting nuclear staining according to immunohistochemistry (IHC) assay. Patients with human epidermal growth factor receptor 2 (HER2) test scores of IHC 3 + (positive) or 2 + (equivocal), and confirmed amplification by fluorescence in situ hybridization (FISH), were classified as HER2 positive; otherwise, they were considered HER2 negative [13]. The pathology results were determined from the most recent data, which could be from the original breast tumor, local recurrent tumor, or metastatic lesions. Adverse effects were documented by physicians using the Common Terminology Criteria for Adverse Events (CTCAE) version 5.0 score [14]. Dose reduction of CDK4/6i was defined as a continuous lower dose than standard dosage on more than two occasions. "Endocrine therapy resistance" was defined as patients who experienced disease relapse under adjuvant endocrine therapy or within 12 months of completing adjuvant endocrine therapy [15]. We also included CDK4/6i resistance, which was defined as the patients treated with CDK4/6i and experiencing disease progression within 12 months of treatment. The time of disease relapse and mortality were recorded and analyzed. Progression-free survival (PFS) was defined as the duration from the date of starting CDK4/6i to the local recurrence, distant metastasis, or death, whichever came first.

### Statistical analysis

All clinicopathological details including age, body mass index (BMI), histology, IHC status, disease status, metastatic site, treatment strategies (endocrine partners, prior chemotherapy, and switching to another CDK4/6i), and safety profiles (dose reduction, regimen changes or discontinuation, adverse effects) for the comparison of the two CDK4/6i were assessed using 1:1 propensity score matching and compared using the χ2 test and Fisher's exact test.

PFS between the palbociclib and ribociclib groups was assessed using Kaplan–Meier survival curve analysis with the log-rank test. Additionally, the Cox proportional hazards model was employed to estimate the risk of relapse and mortality. Statistical significance was defined as *P* < 0.05 for all analyses. All statistical analyses were conducted using R statistical software, version 4.2.1 [16].

## Results

### Study population and patient characteristics

The cohort consisted of 340 patients, with 179 (52.6%) receiving palbociclib and 161 (47.4%) receiving ribociclib. The median follow-up duration was 27.8 months, with 29.4 in the palbociclib and 23.9 in the ribociclib group with an insignificant *P*-value of 0.114. Patient characteristics are detailed in Table 1, demonstrating no significant differences between the two CDK4/6i. After 1:1 propensity score matching by factors such as age, histology, PR status, visceral metastasis, bone-only metastasis, de novo stage IV status, and prior chemotherapy, the matched cohort consisted of 161 patients in both the palbociclib and ribociclib groups (Table 2). The mean age was 61 years, while the palbociclib group had a numerically higher prevalence of over 70 and the ribociclib group had more patients under 50. Most patients had a normal BMI, and the majority had invasive ductal carcinoma (IDC) histology. There were 34.78% and 41.61% of patients with de novo stage IV disease who received palbociclib and ribociclib, respectively. Additionally, 69.57% and 74.53% had visceral metastasis, while 6.83% and 4.35% had local recurrence only, respectively. No significant differences were observed in the distribution of disease statuses and distant metastatic sites between the two groups.

**Table 1:** Clinicopathological characteristics of patients receiving palbociclib or ribociclib before matching (*n* = 340)

Characteristics	CDK4/6i *n* = 340	Palbociclib *n* = 179 (%)	Ribociclib *n* = 161 (%)	*P*-value
Follow-up duration				
Median (months)	27.8	29.4	23.9	0.114
Age				
Mean(range)	60 [27–121]	61 [27–121]	60 [31–100]	0.310
< 50	70 (20.59)	33 (18.44)	37 (22.98)	
50–70	186 (54.71)	99 (55.31)	87 (54.04)	
> = 70	84 (24.71)	47 (26.26)	37 (22.98)	
BMI	23.51	23.71	23.28	0.364
< 18.5	35 (10.29)	15 (8.38)	20 (12.42)	
18.5–24.9	179 (52.64)	100 (55.87)	79 (49.07)	
> = 25.0	106 (31.18)	56 (31.28)	50 (31.06)	
Histology				0.610
IDC	276 (81.18)	142 (79.33)	134 (83.23)	
ILC	22 (6.47)	12 (6.70)	10 (6.21)	
Other/unknown	42 (12.35)	25 (13.97)	17 (10.56)	
IHC status				
ER +	340 (100)	179 (100)	161 (100)	1
PR +	270 (79.41)	144 (80.45)	126 (78.26)	0.640
HER2-	327 (96.18)	170 (94.97)	157 (97.52)	0.423
Ki-67 > = 20	168 (49.41)	85 (47.49)	83 (51.55)	0.552
Disease status				
De novo stage IV	125 (36.76)	58 (32.40)	67 (41.61)	0.100
Local recurrence	21 (6.18)	14 (7.82)	7 (4.35)	0.270
Distant metastasis	319 (93.82)	165 (92.18)	154 (95.65)	
Visceral metastasis	245 (72.06)	125 (69.83)	120 (74.53)	0.399
Site of metastasis				
Bone	200 (58.82)	104 (58.10)	96 (59.63)	0.861
Liver	85 (25.00)	47 (26.26)	38 (23.60)	0.661
Lung	173 (50.88)	87 (48.60)	86 (53.42)	0.437
Brain	16 (4.71)	9 (5.03)	7 (4.35)	0.968
Distant lymph nodes or others metastasis	75 (22.06)	43 (24.02)	32 (19.88)	0.430

**Table 2:** Clinicopathological characteristics of patients receiving palbociclib or ribociclib after propensity score matching (*n* = 322)

Characteristics	CDK4/6i *n* = 322	Palbociclib *n* = 161 (%)	Ribociclib *n* = 161 (%)	*P*-value
Follow-up duration				
Median (months)	28.6	31.0	23.9	0.002
Age				
Mean (range)	61 [27–121]	61 [27–121]	60 [31–100]	0.458
< 50	65 (20.19)	28 (17.39)	37 (22.98)	
50–70	180 (55.90)	93 (57.76)	87 (54.04)	
> = 70	77 (23.91)	40 (24.84)	37 (22.98)	
BMI	23.53	23.66	23.39	0.574
< 18.5	34 (10.56)	14 (8.70)	20 (12.42)	
18.5–24.9	176 (54.66)	194 (58.39)	82 (50.93)	
> = 25.0	106 (32.92)	51 (31.68)	55 (34.16)	
Histology				0.955
IDC	266 (81.18)	132 (79.33)	134 (83.23)	
ILC	21 (6.47)	11 (6.70)	10 (6.21)	
Other/unknown	35 (12.35)	18 (13.97)	17 (10.56)	
IHC status				
ER +	322 (100)	161 (100)	161 (100)	1
PR +	257 (79.81)	131 (81.37)	126 (78.26)	0.579
HER2-	311 (96.58)	154 (95.65)	157 (97.52)	0.502
Ki-67 > = 20	159 (49.38)	76 (47.20)	83 (51.55)	0.504
Disease status				
De novo stage IV	123 (38.20)	56 (34.78)	67 (41.61)	0.251
Local recurrence	18 (5.59)	11 (6.83)	7 (4.35)	0.468
Distant metastasis	304 (94.41)	150 (93.17)	154 (95.65)	
Visceral metastasis	232 (72.05)	112 (69.57)	120 (74.53)	0.384
Site of metastasis				
Bone	186 (57.76)	90 (55.90)	96 (59.63)	0.573
Liver	85 (25.00)	41 (25.47)	38 (23.60)	0.796
Lung	163 (50.62)	77 (47.83)	86 (53.42)	0.373
Brain	16 (4.97)	9 (5.59)	7 (4.35)	0.799
Distant lymph nodes or others metastasis	68 (21.12)	36 (22.36)	32 (19.88)	0.682

### Treatment strategies and safety profiles

The combination of endocrine therapies, including aromatase inhibitors (letrozole, exemestane, and anastrozole), fulvestrant (a selective estrogen receptor degrader), and tamoxifen (a selective estrogen receptor modulator), did not differ significantly between the two groups. Aromatase inhibitors were the most frequently prescribed endocrine partner in both groups. The prevalence of endocrine resistance was approximately 30% in both groups, with no significant difference observed. Notably, the palbociclib group had more patients with prior chemotherapy compared to the ribociclib group before matching (16.7% vs. 8.70%, respectively, *p* < 0.05). The prevalence of prior chemotherapy yielded adequate balance after applied matching on the propensity score (9.94% vs. 8.70%, respectively, *p* = 0.848), as indicated in Table 3.

**Table 3:** The combination of endocrine treatment and the prevalence of endocrine resistance and prior chemotherapy for advanced disease after propensity score matching

	Palbociclib *n* = 161 (%)	Ribociclib *n* = 161 (%)	*P*-value
Endocrine therapy partner (1st line)			
Letrozole	105 (65.22)	118 (41.61)	0.163
Exemestane	12 (7.45)	13 (8.07)	0.835
Anastrozole	11 (6.83)	7 (4.35)	0.332
Fulvestrant	28 (17.39)	24 (14.91)	0.545
Tamoxifen	12 (7.45)	11 (6.83)	0.829
Endocrine resistance	50 (31.06)	46 (28.57)	0.715
Previous chemotherapy	16 (9.94)	14 (8.70)	0.848

^*^p < 0.05

**Fig. 1:**
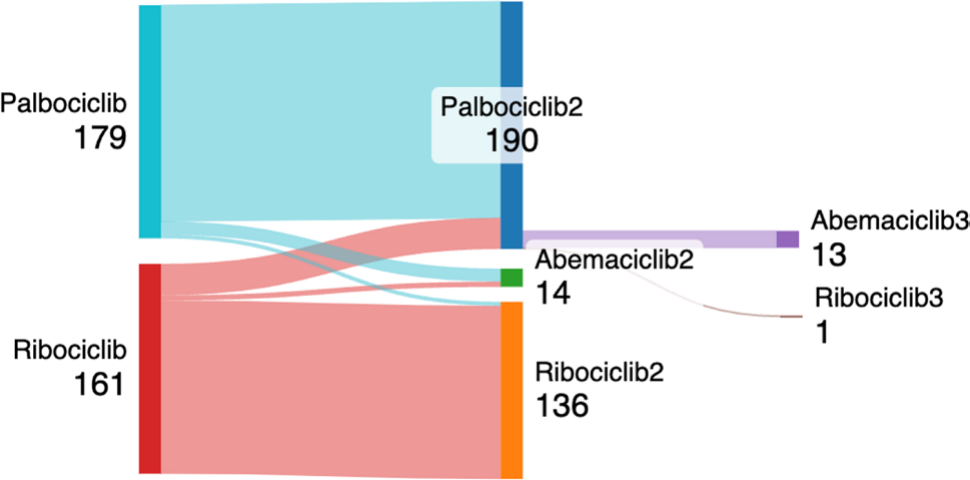
Sankey plot of changing regimens between different types of CDK4/6 inhibitors

Table 4 presents the adverse effects and dose adjustment in the study. Hematotoxicity was the most common adverse effect in both groups. The palbociclib group had significantly higher rates of neutropenia (90.68%), anemia (36.65%), and thrombocytopenia (25.47%) compared to the ribociclib group (76.40%, 24.84%, 10.56%, respectively). In contrast, elevated liver enzymes, skin rash, and QT prolongation occurred more frequently in the ribociclib group (19.25%, 6.21%, 6.83%, respectively) compared to the palbociclib group (6.21%, 1.24%, and 0%). The dose reduction rate was similar in both groups, with 29.81% in the palbociclib group and 24.22% in the ribociclib group. However, patients with palbociclib had a significantly lower rate of regimen changes (10.56%) compared to those with ribociclib (21.12%), which included switching to another CDK4/6 inhibitor or changing the endocrine partner. Three patients switched from palbociclib to ribociclib, 10 patients switched from palbociclib to abemaciclib, 24 patients switched from ribociclib to palbociclib, 4 patients switched from ribociclib to abemaciclib, and only 14 patients changed to a third type of CDK4/6 inhibitor later on (Fig. 1). The most common reason for changing treatment in the palbociclib group was disease progression, while adverse effect was the primary reason in the ribociclib group. Moreover, the palbociclib group had a significantly higher rate of discontinuing CDK4/6i compared to the ribociclib group (70.19% vs. 59.01%, *p* < 0.05), primarily due to disease progression.

**Table 4:** Adverse effects of CDK4/6 inhibitors and causes of dose adjustment

	Palbociclib *n* = 161 (%)	Ribociclib *n* = 161 (%)	*P*-value
Adverse effect (any grade)			
Neutropenia	146 (90.68)	123 (76.40)	* < 0.001
Anemia	59 (36.65)	40 (24.84)	*0.021
Thrombocytopenia	41 (25.47)	17 (10.56)	* < 0.001
Diarrhea	4 (2.48)	7 (4.35)	0.542
Nausea or vomiting	3 (1.86)	11 (6.83)	0.052
Elevated liver enzyme	10 (6.21)	31 (19.25)	* < 0.001
Acute kidney injury	5 (3.11)	8 (4.97)	0.573
Skin rash	2 (1.24)	10 (6.21)	*0.035
QT prolong	0 (0)	11 (6.83)	–
Other	2 (1.24)	2 (1.24)	1.000
Dose reduction	48 (29.81)	39 (24.22)	0.315
Change regimen	17 (10.56)	34 (21.12)	*0.015
To another CDK4/6 inhibitor	13	28	
Change endocrine partner	4	6	
Cause of change regimen			
Adverse effects	1	20	
Progression disease	16	10	
Economic issue	0	4	
Stop CDK4/6 inhibitor use	113 (70.19)	95 (59.01)	*0.048
Adverse effects	6	9	
Progression disease	80	57	
Death	2	4	
Economic issue	16	21	
Loss of following up, unknown	9	4	

^*^p < 0.05

### Treatment efficacy

**Fig. 2:**
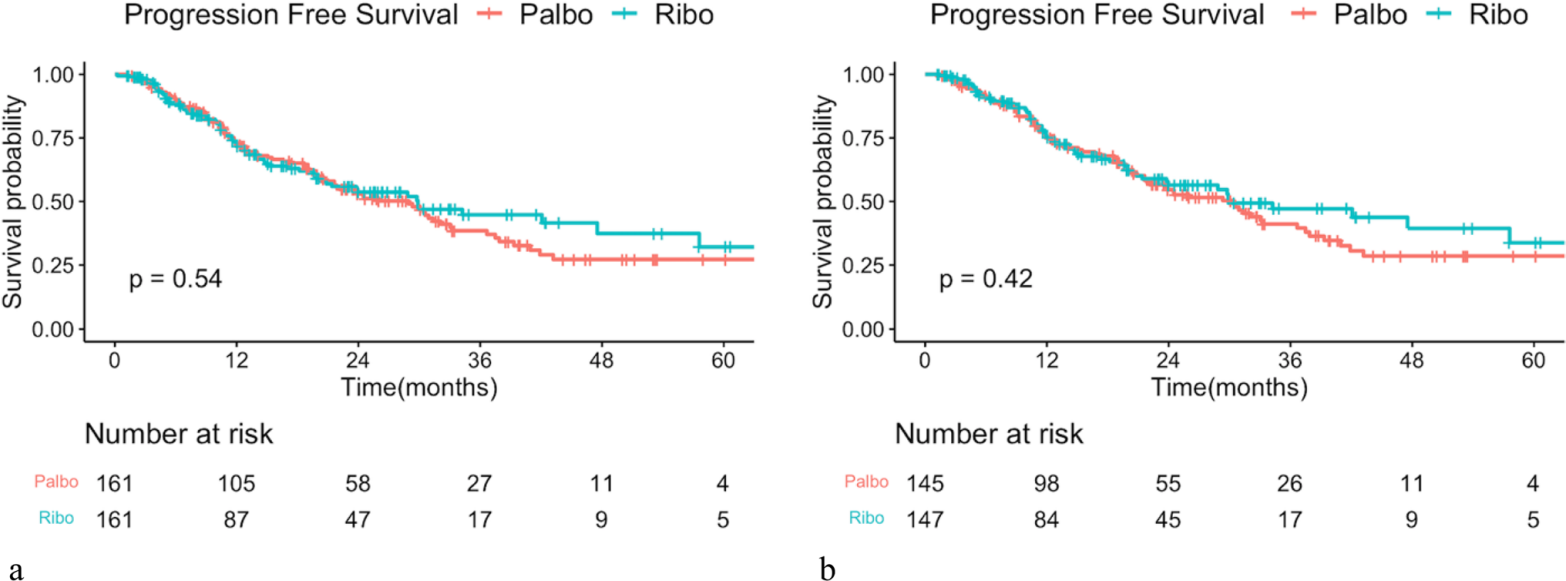
Kaplan–Meier survival curve analysis for progression-free survival (PFS) between patients treated with palbociclib and ribociclib. **a** Whole cohort patients and **b** patients treated in the first-line treatment setting

**Fig. 3:**
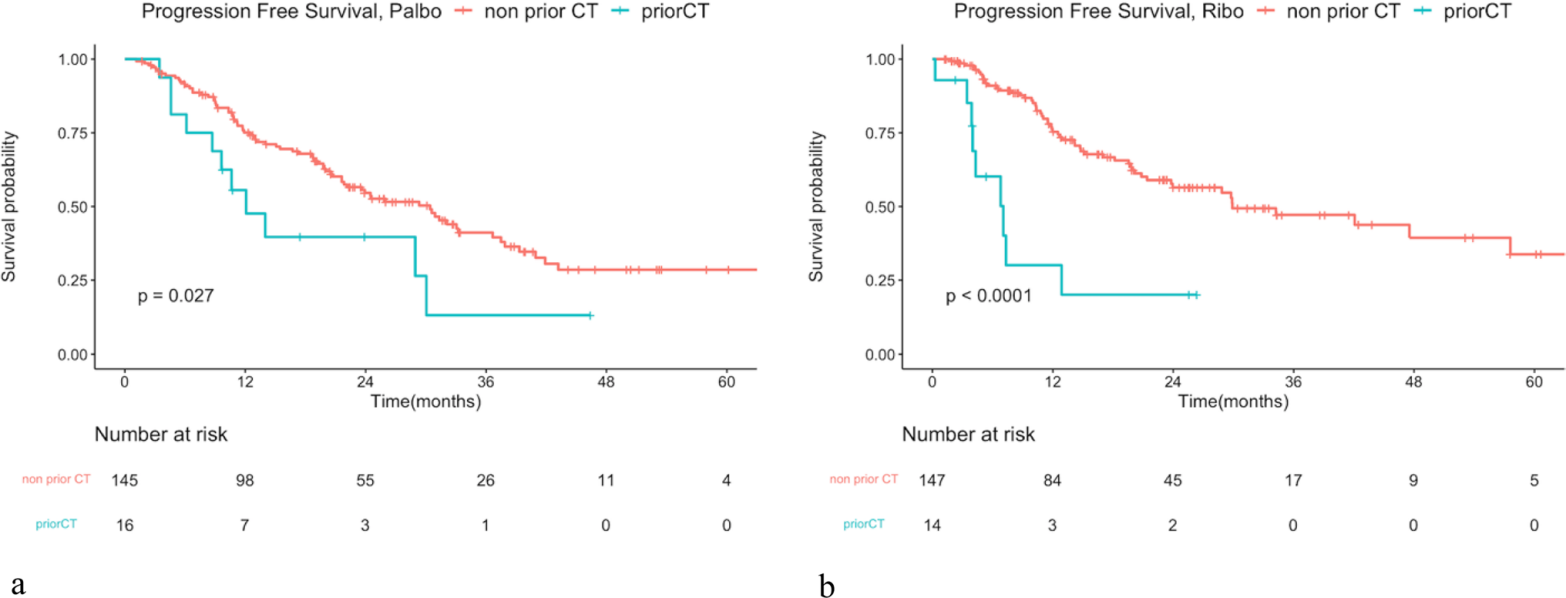
Kaplan–Meier survival curve analysis for progression-free survival (PFS) between patients treated with prior chemotherapy or not. **a** Patients treated with palbociclib. Median PFS (mPFS) in non-prior chemotherapy (CT) and prior CT was 30.42 months versus 12.09 months. Hazard ratio = 2.023, *P* < 0.05. **b** Patients treated with ribociclib. mPFS was 29.89 months in non-prior CT and 7.06 months in prior CT, hazard ratio: 4.276, *P* < 0.05

**Fig. 4:**
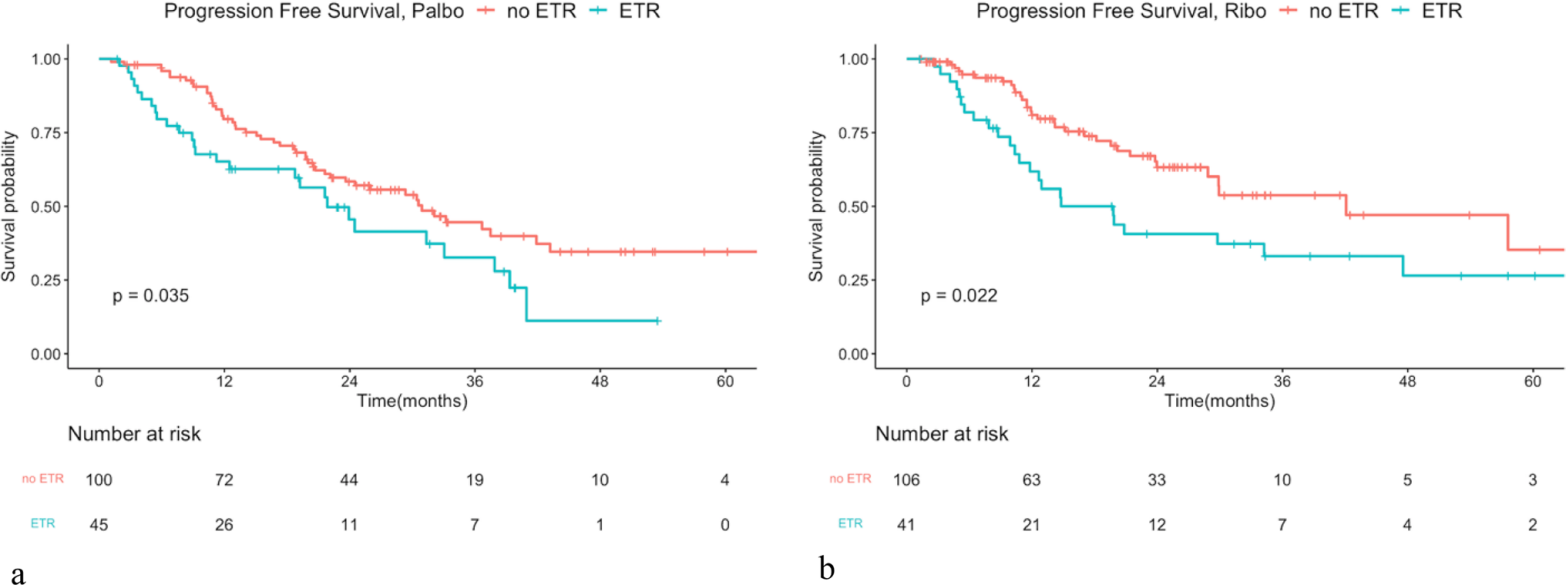
Kaplan–Meier survival curve analysis for PFS between patients treated with endocrine therapy resistance (ETR) or not. **a** Patients treated with palbociclib. mPFS in non-ETR and ETR was 30.91 months versus 21.85 months. Hazard ratio: 1.655, *P* < 0.05. **b** Patients treated with ribociclib. mPFS in non-ETR and ETR was 42.08 months and 19.78 months, hazard ratio: 1.842, *P* < 0.05. *mPFS: medium progression-free survival.

**Fig. 5:**
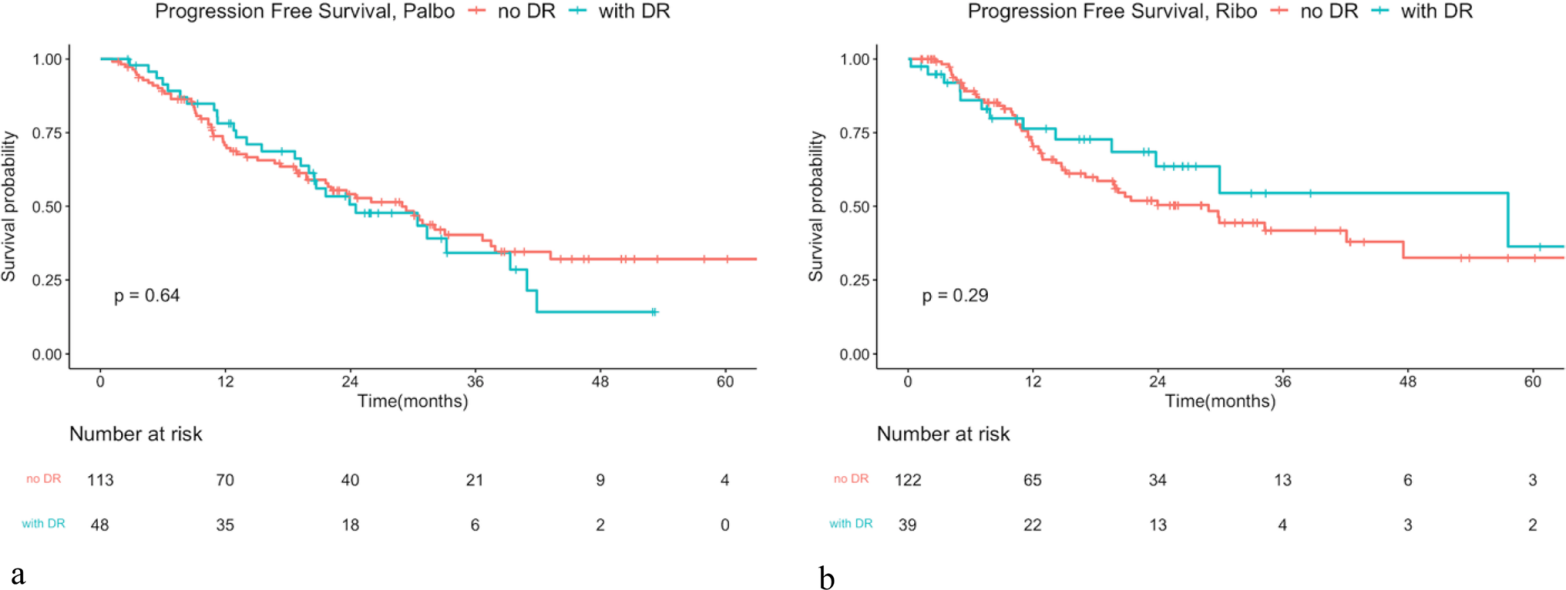
Kaplan–Meier survival curve analysis for PFS between patients with dose reduction (DR) of CDK4/6 inhibitor or not. **a** Patients treated with palbociclib. mPFS in non-dose reduction and dose reduction was 28.94 months versus 24.51 months. Hazard ratio: 1.114, *P* = 0.641. **b** Patients treated with ribociclib. mPFS in non-dose reduction and dose reduction was 28.84 months and 57.59 months, hazard ratio: 0.722, *P* = 0.295. *mPFS: medium progression-free survival.

The median PFS (mPFS) for the full cohort was 29.34 months. Analysis using a Cox regression model showed no significant difference in PFS between patients treated with palbociclib and those with ribociclib. The mPFS was 28.94 months in the palbociclib group and 29.86 in the ribociclib group, with a hazard ratio of 0.905 and a *P*-value of 0.542 (Fig. 2a). Given the significantly higher proportion of patients receiving prior chemotherapy in the palbociclib group compared to the ribociclib group, we conducted a subgroup analysis focusing on patients treated with CDK4/6i as the first-line treatment. The PFS did not differ significantly between palbociclib and ribociclib, with mPFS of 30.42 and 29.89 months, respectively. The hazard ratio was 0.868 with an insignificant *P*-value of 0.419 (Fig. 2b). Patients with prior chemotherapy or endocrine resistance experienced significantly decreased PFS in both groups (Figs. 3 and 4). Furthermore, dose reduction of CDK4/6i showed similar efficacy compared to standard-dose treatment in both groups (Fig. 5).

Table 5 presents the risk of disease relapse as determined by the univariate and multivariate Cox proportional hazards model. In univariate analysis, elevated BMI (HR 0.943, 95% CI 0.907–0.980), bone-only metastasis (HR 0.556, 95% CI 0.350–0.884), and combination with letrozole (HR 0.566, 95% CI 0.408–0.734) were associated with a decreased risk of disease progression, all with *p*-values < 0.05. Conversely, there was a significantly increased risk of disease relapse in patients with elevated HER-2 IHC staining (HR 1.312, 95% CI 1.055–1.631), elevated Ki-67 level (HR 1.018, 95% CI 1.007–1.028), visceral metastasis (HR 1.462, 95% CI 1.009–2.118), liver metastasis (HR 2.663, 95% CI 1.900–3.732), endocrine therapy resistance (HR 1.619, 95% CI 1.166–2.249), and prior chemotherapy treatment (HR 2.713, 95% CI 1.687–4.361), all with *p*-values < 0.05. With multivariate analysis, only elevated Ki-67 level (HR 1.021, 95% CI 1.010–1.033), liver metastasis (HR 2.676, 95% CI 1.759–4.071), endocrine therapy resistance (HR 1.551, 95% CI 1.048–2.297), and prior chemotherapy treatment (HR 2.016, 95% CI 1.110–3.658) were associated with an increased risk of disease progression, all with *p*-values < 0.05.

**Table 5:** Risk factors of disease relapse in breast cancer patients treated with CDK4/6 inhibitor using the univariate and multivariate Cox proportional hazards model

Risk factor	Univariate analysis			Multivariate analysis		
	Hazards ratio	95% confidence interval	*P*-value	Hazards ratio	95% confidence interval	*P*-value
Age	0.998	0.986–1.001	0.765			
BMI	0.943	0.907–0.980	*0.003	0.972	0.930–1.016	0.215
Progesterone receptor status	1.023	0.685–1.527	0.912			
HER2 (IHC)	1.312	1.055–1.631	*0.015	1.052	0.803–1.377	0.713
Ki-67	1.018	1.007–1.028	* < 0.001	1.021	1.010–1.033	* < 0.001
De novo stage IV	0.784	0.560–1.099	0.158			
Visceral metastasis	1.462	1.009–2.118	*0.045			
Bone-only metastasis	0.556	0.350–0.884	*0.013	1.001	0.578–1.734	0.997
Liver metastasis	2.663	1.900–3.732	* < 0.001	2.676	1.759–4.071	* < 0.001
Lung metastasis	1.015	0.739–1.393	0.928			
Brain metastasis	1.207	0.592–2.461	0.605			
Endocrine resistance	1.619	1.166–2.249	*0.004	1.551	1.048–2.297	*0.028
Prior chemotherapy	2.713	1.687–4.361	* < 0.001	2.016	1.110–3.658	*0.021
Dose reduction	0.953	0.666–1.364	0.793			
Hematotoxicity	0.628	0.391–1.008	0.054			
Letrozole as						
Endocrine partner	0.566	0.408–0.734	* < 0.001	0.669	0.434–1.031	0.069

*P < 0.05

Patients treated with CDK4/6i who progressed within 12 months were considered CDK4/6i resistant. Among the 66 patients identified, 36 were treated with palbociclib, and 30 were treated with ribociclib (including 1 patient who switched from ribociclib to palbociclib due to adverse effects), showing resistance to CDK4/6i. Table 6 reveals that patient characteristics did not differ between those with or without CDK4/6i resistance, except for a higher proportion of liver metastasis (39.39% vs. 20.70%), prior chemotherapy treatment (19.70% vs. 6.64%), and endocrine resistance (40.91% vs. 26.95%) in CDK4/6i-resistant patients compared to those without (*p* < 0.05).

**Table 6:** The characteristics of patients with or without CDK4/6 inhibitor resistance

Characteristics	Without CDK4/6i resistance *n* = 256	With CDK4/6i resistance *n* = 66	*P*-value
Age, mean (range)	61 [27–121]	59 [31–100]	0.414
< 50	52 (20.31)	13 (19.70)	
50–70	140 (54.69)	40 (60.61)	
> = 70	64 (25.00)	13 (19.70)	
BMI, mean	23.68	22.92	0.186
Histology			0.346
IDC	209 (81.64)	57 (86.36)	
ILC	16 (6.25)	5 (7.58)	
Other/unknown	31 (12.11)	4 (6.06)	
IHC status			
ER +	256 (100)	66 (100)	1.000
PR +	205 (80.08)	52 (78.79)	0.952
HER2-	250 (97.66)	61 (92.42)	0.087
Ki > = 20	126 (49.22)	33 (50.00)	1.000
Disease status			
De novo stage IV	103 (40.23)	20 (30.30)	0.181
Local recur	18 (7.03)	1 (1.52)	0.139
Distant metastasis	238 (92.97)	65 (98.48)	
Visceral metastasis	179 (69.92)	53 (80.30)	0.128
Site of metastasis			
Bone	145 (56.64)	41 (62.12)	0.507
Liver	53 (20.70)	26 (39.39)	*0.003
Lung	128 (50.00)	35 (53.03)	0.763
Brain	13 (5.07)	3 (4.55)	1.000
Distant lymph nodes or others metastasis	54 (21.09)	20 (30.30)	0.155
Dose reduction	74 (28.91)	13 (19.70)	0.178
Prior chemotherapy	17 (6.64)	13 (19.70)	*0.003
Endocrine resistance	69 (26.95)	27 (40.91)	*0.039
Endocrine therapy partner			
Letrozole	183 (71.49)	41 (62.12)	0.186
Exemestane	21 (8.20)	6 (9.09)	0.805
Anastrozole	16 (6.25)	4 (6.06)	1.000
Fulvestrant	48 (18.75)	15 (22.73)	0.581
Tamoxifen	22 (8.59)	4 (6.06)	0.619

*P < 0.05

### Safety of discontinuation after 2 years of treatment

A subgroup analysis was conducted for 38 patients who discontinued CDK4/6i treatment after 2 years of NHI reimbursement without disease progression. Of these, 17 patients had been treated with palbociclib and 21 with ribociclib. Following discontinuation, 3 ribociclib patients switched to fulvestrant, letrozole, and exemestane, respectively, and experienced disease progression events 231, 276, and 81 days after discontinuing CDK4/6i. Two patients treated with palbociclib switched to exemestane and had disease progression 112 and 210 days after discontinuing CDK4/6i. One patient with ribociclib switched to fulvestrant and died 585 days later (not related to breast cancer), while another palbociclib patient switched to capecitabine and died after 308 days related to breast cancer.

**Fig. 6:**
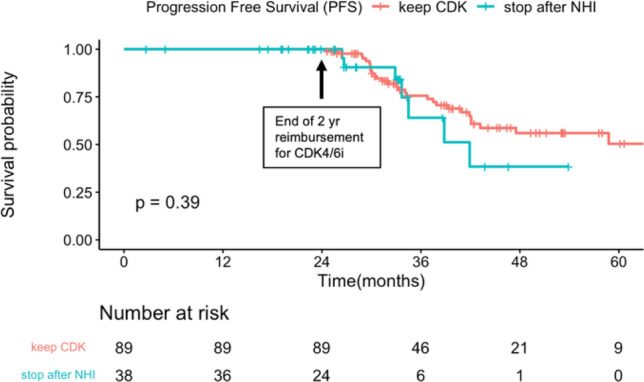
Survival analysis for patients who persisted with treatment and who interrupted treatment after 2 years

We further analyzed a total of 89 patients who continued CDK4/6i for over 2 years with out-of-pocket medication and 38 patients who interrupted CDK4/6i due to economic reasons, to evaluate the impact of early discontinuation. The median follow-up times were 40.8 months and 39.0 months, respectively, with *p*-value < 0.05. Figure 6 illustrates the results of a Kaplan–Meier survival curve, showing no significant difference in PFS between patients who continued treatment and those who interrupted treatment after 2 years (hazard ratio: 1.439, 95% confidence interval: 0.624–3.321, *P* = 0.393).

## Discussion

CDK4/6 inhibitors have emerged as a cornerstone in the treatment of HR-positive metastatic breast cancer over this decade. Three CDK4/6i—palbociclib, ribociclib, and abemaciclib—share a common mechanism of action, inhibiting CDK4/6 to halt the phosphorylation of retinoblastoma protein and arrest the G1 cell cycle phase in tumor cells [17, 18]. These agents have demonstrated similar efficacy in multiple clinical trials [1–3]. Palbociclib, in combination with an aromatase inhibitor, was the first to receive FDA approval for HR-positive/HER2-negative metastatic breast cancer since February 2015, followed by ribociclib and abemaciclib in March 2017 and September 2017, respectively. In Taiwan, National Health Insurance began reimbursing  palbociclib and ribociclib for HR-positive metastatic breast cancer in 2019. Consequently, palbociclib has been the most widely used CDK4/6i, followed by ribociclib, primarily due to the timeline of FDA approvals, marketing, and insurance coverage. In our study, we found no significant difference in patient characteristics between palbociclib and ribociclib. However, there was a numerical predominance of patients aged younger than 50 years treated with ribociclib than palbociclib, while patients older than 70 tended to receive palbociclib. The MONALEESA-7 trial enrolled approximately 27.7% of premenopausal and around 30% of Asian patients, which may have influenced physicians’ choices [19].

Regarding the adverse effects of CDK4/6 inhibitors, hematologic toxicity was the most prevalent in both groups in our cohort. The palbociclib group exhibited significantly higher rates of neutropenia, anemia, and thrombocytopenia compared to the ribociclib group. Conversely, the ribociclib group experienced a higher incidence of nausea, vomiting, elevated liver enzymes, skin rash, and QT prolongation. The adverse events in the palbociclib group were consistent with findings from the PALOMA trial [1]. However, patients in the ribociclib group exhibited significantly lower rates of gastrointestinal symptoms and skin rash compared to those reported in the MONALEESA and CompLEEment-1 trials [20, 21]. These discrepancies may be attributed to potential biases in patient-reported outcomes and medical record documentation. Moreover, palbociclib demonstrated no age-related differences between adverse effect in the PALOMA-2/3 trials in the elderly population. However, the MONALEESA series showed a higher incidence of anemia and gastrointestinal symptoms in patients over 65 years of age treated with ribociclib compared to younger patients. The risk of QT interval prolongation from ribociclib is also a consideration [22]. Despite the safety profiles of both CDK4/6i being established in clinical studies, physicians may lean toward prescribing palbociclib in elderly patients due to these observed differences. Moreover, patients treated with ribociclib, which is associated with a higher prevalence of digestive symptoms, skin rash, and elevated liver enzymes compared to palbociclib, may switch to other CDK4/6 inhibitors due to intolerance of adverse effects or physicians’ and patients’ concerns. In the previous studies, the PALOMA-2 trial failed to demonstrate an overall survival benefit, whereas the MONALEESA-2 trial reported a significant benefit, with an overall survival of 63.9 months [2, 23]. Meanwhile, the further clinical trial of palbociclib, PARSIFAL-Long, determined that the mPFS was 33.2 months, and the OS was 65.4 months, consistent with the OS reported in MONALEESA-2 [24]. This variation underscores the importance of continuously monitoring the evolving patterns of CDK4/6 inhibitor usage in RWD.

Our analysis of the mPFS in patients receiving the first-line CDK4/6i was 30.42 and 29.89 months for the palbociclib and ribociclib groups, respectively. These findings are slightly longer than the results of the PALOMA-2, which reported a mPFS of 27.6, and the MONALEESA-2, which reported a mPFS of 25.3 months. In the aspect of RWD, the P-REALITY X study demonstrated that HR-positive/HER2-negative metastatic breast cancer patients receiving palbociclib plus an aromatase inhibitor (AI) as first-line treatment had a mPFS of 32.6 months in a retrospective comparative analysis from routine practice in the USA [25]. On the other hand, the CompLEEment-1 study evaluated ribociclib plus letrozole in patients with HR-positive/HER2-negative advanced breast cancer, revealing a mPFS of 27.1 months [21]. A single-center study from the UK, focusing on the first-line therapy for HR-positive metastatic breast cancers, also showed similar results, with a mPFS of 27.9 for palbociclib and 29 months for ribociclib [26]. Additionally, a multi-center study in Germany reported a mPFS of 23 months for CDK4/6i in the first-line setting [27]. This study also highlights that patients with a history of extensive prior therapy had a shorter mPFS. Our analysis further demonstrated that patients with prior chemotherapy or endocrine resistance experienced shorter mPFS regardless of whether they received palbociclib or ribociclib. This subset of patients may exhibit aggressive tumor behaviors or have been heavily pretreated, potentially leading to resistance to CDK4/6i.

Regarding the diversity of ethnicity, 14.6% of the patients in PALOMA-2 were Asian, and only 23.9% were from races other than Black (8.1%) and White (68.1%) in the P-REALITY X study [1, 6]. In ribociclib studies, only 8.4% of patients were Asian in MONALEESA-2 and 7% in CompLEEment-1 [20, 21]. A RWD on the use of palbociclib in advanced/metastatic breast cancer in the Asian population from Singapore showed a mPFS of 28.17 months in first-line treatment [9]. Our study cohort consisted entirely of an Asian population, and the outcomes were consistent with previous clinical trials and RWD. These results suggest that there is no significant difference in treatment efficacy between Asian and Western populations. However, there is still a need for more comprehensive RWD on the Asian population to date.

According to previous studies, an increase in BMI is associated with a higher risk of metastasis in obese breast cancer patients. However, they primarily focused on early breast cancers [28, 29]. In our study, we found that a higher BMI was an independent prognostic factor for better outcomes in metastatic HR-positive breast cancer. The mean BMI in our cohort was 23.5 kg/m^2^, which falls within the normal range. In a multi-center national-wide retrospective database study of metastatic breast cancers, it was concluded that overweight and obesity were not associated with poorer outcomes, while being underweighted emerged as an independent adverse prognostic factor [30]. Underweighted patients, which are more common in the Asian region, may be associated with poor health conditions, aggressive disease behavior, or a higher risk of treatment intolerance. Another study focusing on metastatic breast cancer patients treated with CDK4/6i found no difference in mPFS across BMI categories [31]. The correlation between BMI and breast cancer prognosis remains a topic of debate.

Regarding the multivariate Cox regression analysis in our study, only an elevated Ki-67 level, liver metastasis, endocrine resistance, and prior chemotherapy treatment showed a significantly increased risk of disease progression. Additionally, liver metastasis, endocrine resistance, and prior chemotherapy were more prevalent in patients with CDK4/6i resistance compared to those without, suggesting that these patients may have a more aggressive disease status or may be more resistant to treatment due to prior therapies. However, the use of letrozole decreased risk of disease progression in the univariate analysis, which could introduce bias into the study. In clinical practice, patients considered to have endocrine resistance or those receiving higher-line therapy tend to favor fulvestrant over aromatase inhibitors or tamoxifen, based on the findings from the PALOMA-3 and MONALEESA-3 trials [32, 33].

CDK6 plays a crucial role in regulating the proliferation of hematopoietic precursors, leading to hematotoxicity as a major adverse effect of CDK4/6i. Evidence suggests that Asian patients may experience a higher rate of neutropenia and require dose reductions compared to other ethnic populations in the PALOMA series and RWD from Asian countries [34, 35]. Our study also revealed a numerically higher rate of all-grade neutropenia in the palbociclib group (90.7%) compared to 79.5% in the PALOMA-2 study, and in the ribociclib group (76.4%) compared to 74.3% in MONALEESA-2 trial. These findings may be attributed to lower baseline absolute neutrophil counts (ANC) in Asian compared to non-Asian patients [36]. However, the dose reduction rate in the palbociclib group (29.8%) and the ribociclib group (24.2%) in our study was lower than that reported in the PALOMA trial (33.8%), P-REALITY X (41%), MONALEESA-2 (57.5%), and CompLEEment-1 (68.9%) [1, 20, 21, 25] This discrepancy may be due to the inconvenience of changing the dose within the constraints of NHI reimbursement policies, which leads physicians to interrupt treatment rather than to adjust the dose. Previous studies have suggested that neutropenia may be associated with better treatment outcomes, as it is related to the "on-target effect," which may predict efficacy [37–39]. Despite that, this association was not observed in our study, as there was no significant difference in PFS between patients who required dose reductions and those who did not, regardless of being treated with palbociclib or ribociclib. While the safety was confirmed, the impact of dose modification on treatment outcomes remains unclear. Therefore, further evaluation of patients requiring dose reductions is warranted.

Being a novel treatment modality, the economic toxicity of CDK4/6i becomes a significant factor influencing physicians' and patients' decisions. The Taiwan NHI system balances CDK4/6i expenses and patient benefits. Consequently, a decision was made to reimburse two years of CDK4/6i for the 1st and 2nd lines of HR-positive mBC based on the mPFS results from clinical trials. However, there is currently no evidence regarding the prognosis after discontinuation of CDK4/6i treatment after 2 years of reimbursement. In our study, the median follow-up time for the patients who discontinued CDK4/6i due to economic burden was significantly shorter than for patients with continuous treatment of CDK4/6i. This limited follow-up time was insufficient to assess the outcomes of patients treated with interrupted CDK4/6i for up to two years under the NHI reimbursement policy. Understanding these outcomes is crucial for ensuring patients’ safety and evaluating the efficacy of the NHI resource allocation.

This study has several limitations. Firstly, it is based on a retrospective database, which may introduce biases and limitations in data collection. There may be incomplete or inaccurate reporting of adverse effects, especially symptoms relevant to the patient's subjective well-being, such as gastrointestinal symptoms and fatigue without a formal quality-of-life assessment instrument. This might cause an underestimate of adverse effects, compared to the corresponding clinical trials. Also missing data on other facilities' treatments outside our institute, such as previous medical history and self-paid medications, cannot be retrieved from the electrical medical records or the Big Data Center. For some patients, the lack of pathology for primary or metastatic tumors may limit the accuracy of the analysis. Secondly, the sample size in this study was limited, which may affect the generalizability of the findings and subgroup analyses. Thirdly, the follow-up time in this study was limited, which makes it challenging to determine overall survival and assess the long-term effects of CDK4/6i treatment. Lastly, the study did not account for the sequential treatment effects of different CDK4/6 inhibitors and endocrine therapy shifting, which may impact treatment outcomes. Future studies should consider these factors to provide a more comprehensive understanding of the efficacy and safety of CDK4/6i treatments, especially for the Asian population.

## Conclusion

Treatment outcomes for distinct CDK4/6i did not differ significantly, indicating the safety and efficacy of CDK4/6i in the Asian population. Ribociclib and palbociclib showed similar efficacy in terms of PFS in RWD of the Asian population. Patients with prior chemotherapy and endocrine therapy resistance had a negative impact on PFS, yet dose reduction showed no significant difference in PFS between palbociclib and ribociclib.

## Data Availability

No datasets were generated or analyzed during the current study.

## Abbreviations

CDK4/6i: Cyclin-dependent kinase 4/6 inhibitors
HR: Hormone receptor
PSF: Progression-free survival
OS: Overall survival
RCT: Randomized controlled trial
FDA: Food and Drug Administration
RWD: Real-world data
NHI: National Health Insurance
mBC: Metastatic breast cancer
rBC: Relapsed breast cancer
NCCN: National Comprehensive Cancer Network
ESMO: European Society for Medical Oncology
IHC: Immunohistochemistry
HER2: Human epidermal growth factor receptor 2
FISH: Fluorescence in situ hybridization
CTCAE: Common Terminology Criteria for Adverse Events
BMI: Body mass index
IDC: Invasive ductal carcinoma
mPFS: Median PFS
ANC: Absolute neutrophil counts
